# Clinical impact of ventilator-associated pneumonia in patients with the acute respiratory distress syndrome: a retrospective cohort study

**DOI:** 10.1186/s13613-022-00998-7

**Published:** 2022-03-15

**Authors:** Marc Le Pape, Céline Besnard, Camelia Acatrinei, Jérôme Guinard, Maxime Boutrot, Claire Genève, Thierry Boulain, François Barbier

**Affiliations:** 1 Médecine Intensive-Réanimation, Centre Hospitalier Régional d’Orléans, 14, avenue de l’Hôpital, 45100 Orléans, France; 2 Réanimation Chirurgicale, Centre Hospitalier Régional d’Orléans, Orléans, France; 3 Laboratoire de Bactériologie, Pôle de Biopathologies, Centre Hospitalier Régional d’Orléans, Orléans, France; 4 Centre d’Étude des Pathologies Respiratoires (CEPR), INSERM U1100, Université de Tours, Tours, France

**Keywords:** Acute respiratory distress syndrome, Ventilator-associated pneumonia, Intensive care unit, Mechanical ventilation, Hospital-acquired infection, COVID-19, Outcome

## Abstract

**Background:**

The clinical impact and outcomes of ventilator-associated pneumonia (VAP) have been scarcely investigated in patients with the acute respiratory distress syndrome (ARDS).

**Methods:**

Patients admitted over an 18-month period in two intensive care units (ICU) of a university-affiliated hospital and meeting the Berlin criteria for ARDS were retrospectively included. The association between VAP and the probability of death at day 90 (primary endpoint) was appraised through a Cox proportional hazards model handling VAP as a delay entry variable. Secondary endpoints included (i) potential changes in the PaO_2_/FiO_2_ ratio and SOFA score values around VAP (linear mixed modelling), and (ii) mechanical ventilation (MV) duration, numbers of ventilator- and vasopressor-free days at day 28, and length of stay (LOS) in patients with and without VAP (median or absolute risk difference calculation). Subgroup analyses were performed in patients with COVID-19-related ARDS and those with ARDS from other causes.

**Results:**

Among the 336 included patients (101 with COVID-19 and 235 with other ARDS), 176 (52.4%) experienced a first VAP. VAP induced a transient and moderate decline in the PaO_2_/FiO_2_ ratio without increase in SOFA score values. VAP was associated with less ventilator-free days (median difference and 95% CI, − 19 [− 20; − 13.5] days) and vasopressor-free days (− 5 [− 9; − 2] days) at day 28, and longer ICU (+ 13 [+ 9; + 15] days) and hospital (+ 11.5 [+ 7.5; + 17.5] days) LOS. These effects were observed in both subgroups. Overall day-90 mortality rates were 35.8% and 30.0% in patients with and without VAP, respectively (*P* = 0.30). In the whole cohort, VAP (adjusted HR 3.16, 95% CI 2.04–4.89, *P* < 0.0001), the SAPS-2 value at admission, chronic renal disease and an admission for cardiac arrest predicted death at day 90, while the COVID-19 status had no independent impact. When analysed separately, VAP predicted death in non-COVID-19 patients (aHR 3.43, 95% CI 2.11–5.58, *P* < 0.0001) but not in those with COVID-19 (aHR 1.19, 95% CI 0.32–4.49, *P* = 0.80).

**Conclusions:**

VAP is an independent predictor of 90-day mortality in ARDS patients. This condition exerts a limited impact on oxygenation but correlates with extended MV duration, vasoactive support, and LOS.

**Supplementary Information:**

The online version contains supplementary material available at 10.1186/s13613-022-00998-7.

## Introduction

The acute respiratory distress syndrome (ARDS) is a clinical entity resulting from a wide spectrum of infectious or non-infectious conditions and combining bilateral pulmonary infiltrates, altered lung compliance, severe hypoxemia, and histopathological patterns of diffuse alveolar damage [1]. This syndrome may affect up to one fourth of intensive care unit (ICU) patients requiring invasive mechanical ventilation (MV) and is linked with hospital mortality rates ranging from 35 to 45%, poor long-term functional prognosis, and substantial utilization of healthcare resources [2–4].

Patients with ARDS appear at high risk for ventilator-associated pneumonia (VAP) due to protracted MV exposure, impaired innate as well as adaptative lung immunity, and dysregulation of the respiratory microbiota [5]. In the general population of intubated patients, the occurrence of VAP is associated with delayed MV weaning and extended ICU length of stay (LOS); however, the attributable mortality of this condition is still debated, varying from ~ 1% to ~ 13% in the available literature [6, 7]. Such data are scarce in the specific subgroup of patients with ARDS and mainly come from studies conducted before the implementation of current policies for VAP prevention and lung protection [8–12].

A vast majority of patients receiving MV for severe coronavirus disease 2019 (COVID-19) meet the Berlin criteria for ARDS [13–16]. These subjects are at increased hazard of VAP when compared to mixed (i.e., ARDS and no ARDS) and/or historical cohorts of non-COVID-19 patients [15, 17–19]. Yet, to the best of our knowledge, whether the epidemiological features, clinical impact and outcomes of VAP differ between patients with COVID-19-related ARDS and those with ARDS from other aetiologies has not been specifically investigated.

The objective of this study was to appraise the clinical impact and outcomes of a first VAP episode in a contemporary cohort of patients with ARDS. Day-90 mortality was the primary endpoint. Secondary endpoints included changes in the arterial partial pressure of oxygen/fraction of inspired oxygen (PaO_2_/FiO_2_) ratio and Sequential Organ Failure Assessment (SOFA) score values around VAP, MV duration, number of ventilator-free days and vasopressor-free days at day 28, and ICU and hospital LOS. These endpoints were investigated on the whole study cohort then separately in patients with COVID-19-related ARDS and those with ARDS from other causes.

## Patients and methods

### Study design and setting

This retrospective cohort study was conducted over an 18-month period (April 1, 2019–September 30, 2020) in the 32-bed medical ICU and the 30-bed surgical ICU of a 1100-bed tertiary care and university-affiliated hospital in France (see the Additional file 1 for details). In these ICUs, all intubated patients meeting the criteria for ARDS are managed with protective ventilatory settings, continuous infusion of neuromuscular blocking agents (NMBA) and routine prone positioning (PP) ≥ 16 h per day at the early phase of moderate-to-severe ARDS (PaO_2_/FiO_2_ < 150 mmHg), nitric oxide inhalation in case of severe hypoxemia and/or ARDS-related *acute cor pulmonale*, and the use of veno-venous extra-corporeal membrane oxygenation (ECMO-VV) for eligible patients with refractory hypoxemia (PaO_2_/FiO_2_ < 60–80 mmHg) and/or a plateau pressure > 30 cmH_2_O despite the implementation of the aforementioned protective ventilatory settings and procedures, in accordance with current guidelines [20]. Corticosteroids are considered on a case-by-case basis in patients with early or late ARDS [21]. Dexamethasone was routinely administered to COVID-19 patients from July 2020 [22]. Bundles for VAP prevention and policies for VAP diagnosis and treatment are exposed in the Additional file 1.

### Patient recruitment, data collection and definitions

All patients admitted over the inclusion period and intubated for ≥ 3 calendar days were identified using coding registries then screened for the Berlin criteria of ARDS through medical chart reviewing: those presenting these criteria for ≥ 2 calendar days were enrolled in the study cohort [23]. Variables exposed in the tables were extracted from (i) computerized medical charts including automatedly implemented biological, MV and monitoring data (ICCA software, Philips, Amsterdam, The Netherlands) and (ii) the microbiology laboratory database.

All episodes of VAP prospectively diagnosed by attending physicians and mentioned in the medical charts were retrospectively evaluated and retained for analyses provided that they fulfilled the following criteria: (i) new or progressive persistent pulmonary infiltrates on chest X-ray combined with (ii) purulent tracheal secretions, (iii) fever or hypothermia (body temperature ≥ 38.5 °C or ≤ 36.5 °C, respectively) and/or leukocytosis or leukopenia (white blood cells count ≥ 10.4 mL or ≤ 4  × 10.3 mL, respectively), and (iv) a positive quantitative lower respiratory tract sample (endotracheal aspirate [ETA] ≥ 10.5 colony-forming unit [CFU]/mL, broncho-alveolar lavage [BAL] fluid ≥ 10.4 CFU/mL or plugged telescopic catheter [PTC] ≥ 10.3 CFU/mL) in patients with prior MV duration ≥ 3 calendar days. This definition was based on current guidelines [24–26]. Ambiguous cases were solved by consensus among the investigators. VAP without microbiological documentation were discarded. Ventilator-associated tracheobronchitis (VAT) episodes were not studied in this work.

COVID-19 was documented through detection of severe acute respiratory syndrome coronavirus-2 (SARS-CoV-2) in nasopharyngeal or lower respiratory tract sample using real-time polymerase chain reaction. Adequate antimicrobial therapy was defined as the administration of at least one agent with *in-vitro* activity on the causative pathogens. The PaO_2_/FiO_2_ ratio was obtained from the results of blood gas collected at least once a day in every patient with ARDS in the participating ICUs—in cases of multiple blood gas samples collected on a given day, the worst daily PaO_2_/FiO_2_ ratio value was analysed. The SOFA score was calculated using the biological values of the corresponding day or, when not measured, those from the closest day. Ventilator-free days and vasopressor-free days at day 28 were, respectively, defined as the total number of calendar days without invasive MV and vasoactive support over the first 28 days following intubation (day 0), with a zero-value attributed to patients deceased during this timeframe [27].

The study protocol was approved on November 27th, 2020 by the Ethical committee of the French Society of Intensive Care (CE-SRLF-20-84). Results of this study are reported according to the STROBE guidelines [28]. Missing values are exposed in the Additional file 1: Table S1.

### Statistical analyses

Data are expressed as median (interquartile range) for continuous variables and number (%) for categorical variables, unless otherwise indicated. Patient characteristics were compared using the Mann–Whitney *U* test for continuous variables and the Fisher’s exact test or χ^2^ test for categorical variables, as appropriate. Missing values were not imputed, since all analysed variables were available for ≥ 98% of patients.

The relationship between the cause of ARDS (that is, COVID-19 versus others) and the cumulative likelihood of VAP over time was appraised through the Gray test handling MV weaning and death as competing events, with calculation of sub-distribution hazard ratio (HR) and 95% confidence interval (CI).

Temporal changes in the PaO_2_/FiO_2_ ratio and SOFA score values around the day of VAP diagnosis (i.e., from Day_VAP_ − 2 [D_VAP_ − 2] to D_VAP_ + 7) were analyzed by linear mixed modelling after preliminary checking of the normal distribution of these variables through inspection of density plots and quantile–quantile plots. For this analysis, ARDS aetiologies (COVID-19 versus others), time-points and the interaction term “group by time” were entered as fixed-effect variables, while patients were entered as random-effect variables with correlated intercept and slope. Post-hoc comparisons of estimated marginal means with 95% CI were adjusted by the Tukey method.

Outcome variables (that is, numbers of ventilator-free and vasopressor-free days at day 28, ICU and hospital LOS, and in-ICU, in-hospital and day-90 mortality rates) were compared between patients with and without VAP through the calculation of median or absolute risk differences with corresponding 95% CI. The Kruskal–Wallis rank sum test was used to assess differences in the number of ventilator-free days between COVID-19 and non-COVID-19 patients and/or according to whether patients developed VAP within the first 28 days or not and/or were discharged alive from the ICU or not. The relationship between the occurrence of VAP and the cumulative likelihood of MW weaning over time, presented as sub-distribution HR and 95% CI, was evaluated through the Gray test handling VAP as a delay entry variable and death as a competing event.

The associations of VAP occurrence and COVID-19 status with the probability of death at day 90 were studied in the framework of a Cox proportional hazards model with robust variance and adjustment for baseline covariables linked with death in bivariable analysis (*P* < 0.2). VAP was handled as a delay entry variable [29]. VAP and COVID-19 as the cause of ARDS were forced in the model. Collinearity was checked by calculation of the variance inflation factor for each other variable introduced in the model. Potential violation of the proportional assumption was appraised by examining the Schoenfeld residual plots. For patients discharged alive from the hospital but lost to follow-up before day 90, the vital status was censored at the date of last available information. The cumulative probability of survival after the onset of VAP was compared between the two subgroups using the log-rank test.

All analyses were conducted using the R software version 3.5.1 (http://www.R-project.org). Two-tailed *P* values < 0.05 were considered statistically significant.

## Results

### Study population

A total of 336 patients were enrolled in the study, including 101 with COVID-19-related ARDS and 235 with ARDS from other causes (Additional file 1: Fig. S1). Among the latter, 152 (64.7%) were admitted between April 2019 and February 2020 (that is, before the beginning of the pandemic) and the remaining 83 (35.3%) between March and September 2020. The characteristics of the study population are summarized in Table 1 and fully exposed in Additional file 1: Table S1. Bacterial or non-SARS-CoV-2 viral pneumonia, aspiration and extra-pulmonary sepsis were the leading causes of ARDS in non-COVID-19 patients. ARDS was classified as mild, moderate and severe in 50 (14.9%), 116 (34.5%) and 170 (50.6%) patients, respectively—this distribution was similar in patients with and without COVID-19.

**Table 1:** Characteristics of the study population

Characteristics	All patients with ARDS (n = 336)	Patients with COVID-19-related ARDS (n = 101)	Patients with ARDS from other causes (n = 235)	*P* value
Male sex	247 (73.5)	73 (72.3)	174 (74.0)	0.79
Age, years	67 (57–74)	67 (58–72)	66 (55–74)	0.73
BMI, kg.m^−2^	28.5 (25.0–32.6)	29.4 (26.1–32.1)	27.8 (24.1–32.9)	0.13
Immune deficiency	53 (15.8)	11 (10.9)	42 (17.9)	0.14
SAPS 2 at ICU admission	50 (38–67)	40 (33–49)	56 (43–71)	< 0.0001
SOFA score at ICU admission	8 (5–11)	5 (3–8)	9 (7–12)	< 0.0001
ARDS aetiology				
COVID-19^a^	101 (30.0)	101 (100)	–	NA
Bacterial or non-SARS-CoV-2 viral pneumonia	106 (31.6)	–	106 (45.1)	
Aspiration	60 (17.9)	–	60 (25.5)	
Extra-pulmonary sepsis	45 (13.4)	–	45 (19.1)	
Miscellaneous	24 (7.1)	–	24 (10.2)	
ARDS and MV characteristics^b^				
Lowest Vt, mL kg^−1^ (PBW) Highest PEEP, cmH_2_O Highest plateau pressure, cmH_2_O Highest driving pressure, cmH_2_O Lowest PaO_2_/FiO_2_ ratio, mmHg Highest PaCO_2_, mmHg Lowest pH	6.1 (5.8–6.6) 10 (7–13) 24 (20–27) 13 (10–16) 100 (74–163) 44 (40–52) 7.36 (7.25–7.4)	6.0 (5.8–6.3) 12 (11–14) 26 (24–28) 13 (11–15) 91 (76–138) 43 (38–49) 7.36 (7.30–7.42)	6.1 (5.7–6.8) 8 (6–12) 23 (18–26) 13 (10–16) 105 (74–172) 46 (40–55) 7.35 (7.22–7.39)	0.02 < 0.0001 < 0.0001 0.91 0.17 0.0005 < 0.0001
ARDS classification (Berlin definition)^b^ Mild	50 (14.9)	11 (10.9)	39 (16.6)	0.09
Moderate Severe	116 (34.5) 170 (50.6)	30 (29.7) 60 (59.4)	86 (36.6) 110 (46.8)	
ARDS-targeted therapies				
Prone positioning Number of days Nitric oxide inhalation Neuromuscular blocking agents	127 (37.8) 5 (2–11) 90 (26.8) 209 (62.2)	75 (74.3) 8 (3–16) 46 (45.5) 86 (85.1)	52 (22.1) 2 (1–5) 44 (19.1) 123 (52.3)	< 0.0001 < 0.0001 < 0.0001 < 0.0001
Organ support during the ICU stay				
Invasive MV duration, overall, days Vasopressors Renal replacement therapy VV-ECMO VA-ECMO	11 (7–20) 280 (83.3) 85 (25.3) 15 (4.5) 7 (2.1)	17 (10–26) 82 (81.2) 23 (22.8) 7 (6.9) 0	9 (6–16) 198 (84.2) 62 (26.4) 8 (3.4) 7 (3.0)	< 0.0001 0.52 0.58 0.16 0.11
Ventilator-associated pneumonia				
First episode Prior MV duration, days More than one episode	176 (52.4) 7 (4–11) 59 (17.6)	69 (68.3) 9 (8–13) 35 (34.6)	107 (45.5) 6 (4–10) 24 (10.2)	0.0001 0.01 < 0.0001

Data are expressed as number (%) or median (interquartile range)

*ARDS* acute respiratory distress syndrome, *COVID-19* coronavirus disease 2019, *BMI* body mass index, *COPD* chronic obstructive pulmonary disease, *ICU* intensive care unit, *LOS* length of stay, *SAPS 2* simplified acute physiology score 2, *SOFA* sepsis-related organ failure assessment, *MV* mechanical ventilation, *Vt* tidal volume, *PBW* predicted body weight, *PEEP* positive end-expiratory pressure, *VV/VA-ECMO* veno-venous/veno-arterial extracorporeal membrane oxygenation

^a^Including 8 cases (7.9%) with bacterial and/or viral co-infection

^b^First day with ARDS criteria

Full characteristics of the study population are provided in Additional file 1: Table S1

### Incidence and clinical features of VAP

Overall, a first episode of VAP was documented in 176 patients (52.4%) after a median of 7 (4–11) days of MV. Factors associated with the occurrence of VAP are exposed in Table 2. The hazard of VAP was higher in COVID-19 patients (cumulative incidence, 69 out of 101, 68.3%) than in those with ARDS from other causes (107 out of 235, 45.5%) after adjustment on the competing risks of extubation and death (sub-distribution HR 1.64, 95% CI 1.23–2.18, *P* = 0.0007) (Additional file 1: Fig. S2). The crude prevalence of VAP in patients with non-COVID-19-related ARDS remained stable after the beginning of the pandemic (67/152 [44.1%] before March 2020 and 40/83 patients [48.2%] from March 2020, *P* = 0.58).

**Table 2:** Factors associated with the occurrence of VAP

	Patients with VAP (n = 176)	Patients without VAP (n = 160)	*P* value
Male sex	141 (80.1)	106 (66.2)	0.004
Age, years	66 (57–73)	68 (57–74)	0.38
BMI, kg m^−2^	28.5 (25.0–31.6)	28.5 (24.9–33.1)	0.64
Past or current smoking	70 (39.8)	62 (38.7)	0.91
Chronic diseases			
Diabetes mellitus COPD Respiratory, others Cardiac Hepatic Renal Immune deficiency Solid or haematological malignancy Others	49 (27.8) 19 (10.8) 28 (15.9) 55 (31.2) 17 (9.7) 11 (6.2) 20 (11.4) 11 (6.2) 9 (5.1)	46 (28.7) 21 (13.1) 17 (10.6) 44 (27.5) 12 (7.5) 17 (10.6) 33 (20.6) 23 (14.4) 12 (7.5)	0.90 0.61 0.20 0.47 0.56 0.17 0.02 0.02 0.38
ARDS aetiology			
COVID-19 Other causes	69 (39.2) 107 (60.8)	32 (20.0) 128 (80.0)	0.0001
SAPS 2 at ICU admission	49 (38–66)	51 (38–67)	0.60
SOFA score at ICU admission	8 (5–10)	8 (6–11)	0.11
Lymphocyte count, mm^−3^			
ICU admission Day 7 Day 14	760 (500–1220) 775 (487–1202) 970 (637–1407)	705 (400–1152) 810 (520–1210) 1000 (620–1505)	0.18 0.78 0.73
ARDS classification (Berlin definition)^a^			
Mild Moderate Severe	23 (13.1) 57 (32.4) 96 (54.5)	27 (16.9) 59 (36.9) 74 (46.2)	0.29
ARDS-targeted therapies			
Prone positioning Number of days Neuromuscular blocking agents	92 (52.3) 7 (2–13) 128 (72.7)	35 (21.9) 2 (1–6) 81 (50.6)	< 0.0001 0.0006 < 0.0001
Corticosteroids (all pooled)^b^	81 (46.0)	88 (55.0)	0.10
Proton pump inhibitor^b^	161 (91.5)	143 (89.4)	0.58
Intra-hospital transport^b^	107 (60.8)	77 (48.1)	0.02
Life-sustaining therapies			
Invasive MV duration, overall, days Vasopressors Renal replacement therapy VA-ECMO VV-ECMO	17 (12–29) 157 (89.2) 52 (29.5) 4 (2.3) 13 (7.4)	7 (5–10) 123 (76.9) 33 (20.6) 3 (1.9) 2 (1.2)	< 0.0001 0.003 0.08 1 0.007

Data are expressed as number (%) or median (interquartile range)

*VAP* ventilator-associated pneumonia, *ARDS* acute respiratory distress syndrome, *COVID-19* coronavirus disease 2019, *BMI* body mass index, *COPD* chronic obstructive pulmonary disease, *ICU* intensive care unit, *LOS* length of stay, *SAPS 2* simplified acute physiology score 2, *SOFA* sepsis-related organ failure assessment, *MV* mechanical ventilation, *VA/VV-ECMO* veno-arterial/veno-venous extracorporeal membrane oxygenation

^a^First day with ARDS criteria

^b^Before the occurrence of first ventilator-associated pneumonia (VAP), or during the whole ICU stay in patients without VAP

The microbiological documentation of VAP was obtained through ETA, BAL and PTC in 135 (76.7%), 27 (15.3%) and 14 (8.0%) patients, respectively. Prior antimicrobial exposure and the distribution of pathogens responsible for VAP are exposed in Additional file 1: Table S2. Enterobacterales (60.8%), *Pseudomonas aeruginosa* (18.2%), *Staphylococcus aureus* (11.4%) and *Stenotrophomonas maltophilia* (10.8%) were the most common causative microorganisms. One hundred and twenty patients (68.2%) received adequate antimicrobial therapy within 24 h following the diagnosis of VAP (COVID-19 patients versus others, 46 [66.7%] versus 74 [69.2%], *P* = 0.74).

### Primary study endpoint

**Fig. 1:**
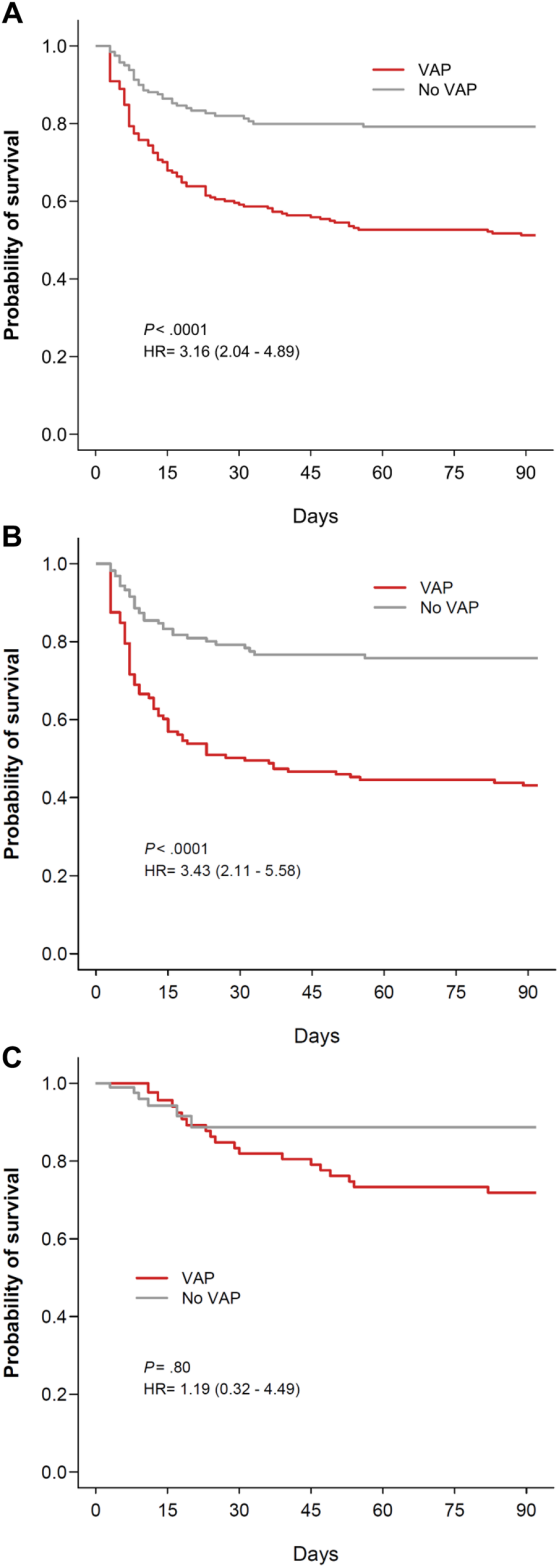
Cumulative likelihood of survival over time in patients with and without VAP. *VAP* ventilator-associated pneumonia, *HR* hazard ratio (indicated with 95% confidence interval). Day 0 indicates the date of intubation. Panel **A**, all patients with acute respiratory distress syndrome (ARDS); Panel **B**, patients with non-coronavirus disease 2019 (COVID-19)-related ARDS; Panel **C**, patients with COVID-19-related ARDS

Crude day-90 mortality rates did not differ between patients with and without VAP (65 [35.8%] versus 48 [30.0%] deceased patients, mean difference and 95% CI, 5.8% [− 4.3%; 15.6%], *P* = 0.30) (Table 3). After adjustment on potential confounders, VAP (adjusted HR [aHR] 3.16, 95% CI 2.04–4.89, *P* < 0.0001), the SAPS 2 value at ICU admission (aHR per 1-point increase 1.02, 1.00–1.03, *P* = 0.005), chronic renal disease (aHR 2.11, 1.10–4.05, *P* = 0.02) and cardiac arrest as the mean reason for ICU admission (aHR 2.00, 1.02–3.92, *P* = 0.04) predicted death at day 90, while the COVID-19 status had no independent effect (aHR 0.94, 0.54–1.66, *P* = 0.84) (Additional file 1: Table S3; Fig. 1). The association between VAP and day-90 mortality was not modified when forcing prone positioning and steroid use during the ICU stay into the model (aHR, 2.67, 1.72–4.14, *P* < 0.0001) (Additional file 1: Table S3). However, when applying the same model separately to both subgroups, the occurrence of VAP was an independent predictor of death at day 90 in patients with non-COVID-19-related ARDS (aHR 3.43, 95% CI 2.11–5.58, *P* ≤ 0.0001) but not in those with COVID-19-related ARDS (aHR 1.19, 95% CI 0.32–4.49, *P* = 0.80) (Fig. 1). The cumulative probability of survival after the onset of VAP was higher in COVID-19 patients than in those with other ARDS (log-rank test, *P* = 0.02) (Additional file 1: Fig. S3).

**Table 3:** Main outcome measures

Outcome measures	All patients with ARDS			Patients with COVID-19-related ARDS			Patients with ARDS from other causes		
	VAP (n = 176)	No VAP (n = 160)	Difference (95% CI)	VAP (n = 69)	No VAP (n = 32)	Difference (95% CI)	VAP (n = 107)	No VAP (n = 128)	Difference (95% CI)
MV duration, days	17 (12–29)	7 (5–10)	10 (8; 12)	22 (17–33)	8 (7–13)	14 (10; 17.5)	15 (9–24)	7 (5–10)	8 (6; 10)
Ventilator-free days at day 28^1^ All patients ICU survivors	0 (0–12) 11 (0–15)	19 (0–22) 21 (18–23)	− 19 (− 20; − 13.5) − 10 (− 13; − 9)	2 (0–11) ^b^ 7 (0–11.5)	19 (11–21) 20 (16.5–21)	− 17 (− 20; − 9.5) − 13 (− 17; − 9)	0 (0–14) ^b^ 12 (2–17)	19 (0–22) 21 (19–23)	− 19 (− 20; − 10) − 9 (− 11; − 6)
Vasopressor-free days at day 28 ^a^	18 (0–24)	23 (0–27)	− 5 (− 9; − 2)	18 (10–25)	25 (20.5–28)	− 7 (− 11; − 1)	16 (0–24)	23 (0–26)	− 7 (− 15; − 2)
ICU LOS, days ^a^	23 (16–36)	10 (8–14)	13 (9; 15)	27 (19–41)	11 (10–16)	15 (11.5; 21.5)	19 (13–28)	10 (8–14)	9 (7; 13)
Hospital LOS, days ^a^	32 (21–48)	21 (12–31)	11.5 (7.5; 17.5)	37 (25–50)	21 (16–28)	15.5 (11; 24)	29 (17–47)	20 (11–34)	8.5 (2; 15)
In-ICU mortality	59 (33.5)	43 (26.9)	6.6 (− 3.2; 16.2)	18 (26.1)	6 (18.7)	7.3 (− 11.5; 22.4)	41 (38.3)	37 (28.9)	9.4 (− 2.6; 21.3)
In-hospital mortality	63 (35.8)	45 (28.1)	7.7 (− 2.3; 17.4)	18 (26.1)	6 (18.7)	7.3 (− 11.5; 22.4)	45 (42.1)	39 (30.5)	11.6 (− 0.7; 23.5)
Mortality at day 90 ^c^	63 (35.8)	48 (30.0)	5.8 (− 4.3; 15.6)	18 (26.1)	6 (18.7)	7.3 (− 11.5; 22.4)	45 (42.1)	42 (32.8)	9.2 (− 3.1; 21.3)

Data are expressed as number (%) or median (interquartile range), with median difference for continuous variable and absolute risk difference for ICU and in-hospital mortality rates

*ARDS* acute respiratory distress syndrome, *VAP* ventilator-associated pneumonia, *CI* confidence interval, *COVID-19* coronavirus disease 2019, *MV* mechanical ventilation, *ICU* intensive care unit, *LOS* length of stay

^a^*P* < 0.001 for the comparison between COVID-19 and non-COVID-19 patients and/or according to whether patients developed VAP within the first 28 days or not

^b^Median difference (95% CI), 2 (− 8; 6) days

^c^Nine patients were lost to follow-up at day 90 (5 patients with VAP and 4 patients without VAP)

### Secondary study endpoints

**Fig. 2:**
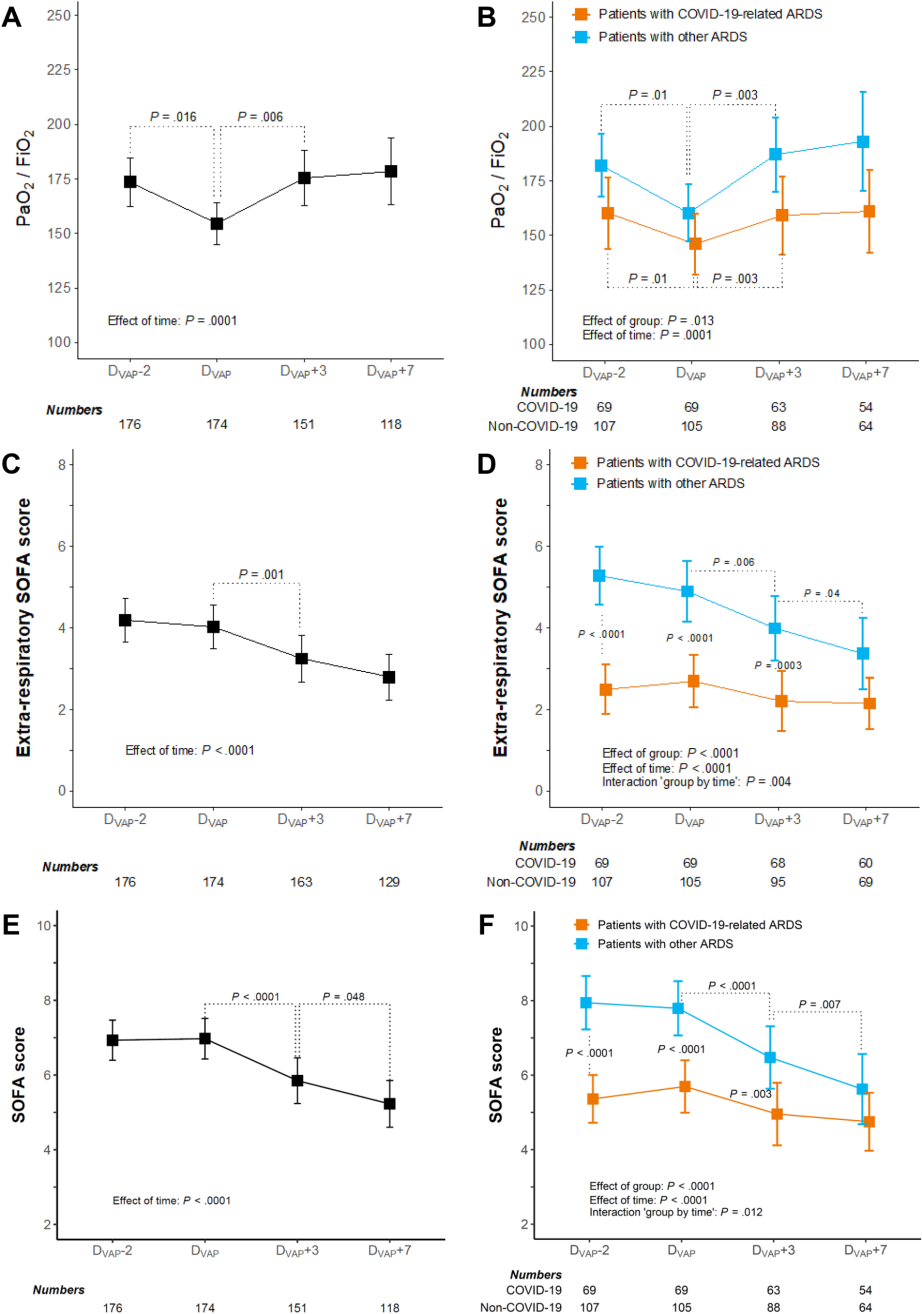
Trends in PaO_2_/FiO_2_ ratio, extra-respiratory SOFA score values and total SOFA score values in patients with VAP. *VAP* ventilator-associated pneumonia, *ARDS* acute respiratory distress syndrome, *COVID-19* coronavirus disease 2019, *SOFA* sepsis-related organ failure assessment. Panels **A**, **C** and **E**, all patients with ARDS; panels **B**, **D** and **F**, patients with COVID-19-related ARDS versus patients with ARDS from other causes

The PaO_2_/FiO_2_ ratio declined from 174 (162–185) mmHg at D_VAP_ − 2 to 155 (144–166) mmHg at D_VAP_ then re-increased to 177 (165–188) mmHg at D_VAP_ + 3 and 181 (168–194) mmHg and D_VAP_ + 7 (estimated marginal means and 95% CI, *P* < 0.05 for the comparison with D_VAP_ at each other timepoint) (Fig. 2A). Time courses of the PaO_2_/FiO_2_ ratio around D_VAP_ did not differ between the two subgroups though absolute values were lower in COVID-19 patients (*P* = 0.01 at each timepoint) (Fig. 2B). Of note, the level of PEEP was similar and remained unchanged around D_VAP_ in both subgroups (median value at D_VAP_, overall, 10 [8–13] cmH_2_O) (Additional file 1: Fig. S4).

The extra-respiratory and total SOFA score values did not evolve significantly between D_VAP_ − 2 and D_VAP_ then decreased after D_VAP_ in non-COVID-19 patients (Fig. 2D, F). In COVID-19 patients, no variation was observed in the extra-respiratory and total SOFA score values around D_VAP_; these values were significantly lower than those observed in patients with ARDS from other causes.

**Fig. 3:**
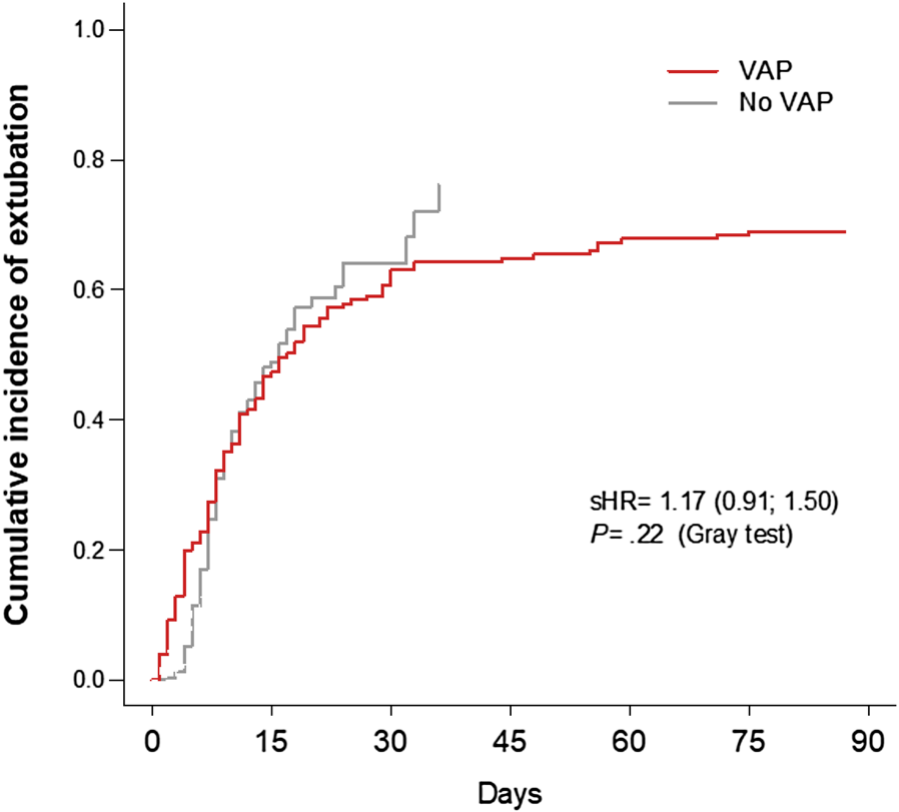
Cumulative likelihood of MV weaning in patients with and without VAP. *MV* mechanical ventilation, *VAP* ventilator-associated pneumonia, *sHR* cause-specific hazard ratio (indicated with 95% confidence interval). day 0 indicates the date of intubation. Note that the curve of the no-VAP subgroup ends at day 36 of MV, since all patients without VAP had been extubated or had died at this time. For the VAP subgroup, the curve ends at day 89 of MV for the same reasons

Overall, patients with VAP experienced less ventilator-free days at day 28 than those not developing this condition (median difference and 95% CI, − 19 [− 20; − 13.5] days), with a similar difference in both subgroups (Table 3). After adjustment on the competing risk of death, the cumulative likelihood of MV weaning differed neither between patients with and without VAP (sub-distribution HR 1.17, 95% CI 0.91–1.50, *P* = 0.22) (Fig. 3) nor according to the COVID-19 status in patients with VAP (sub-distribution HR 1.10, 95% CI 0.75–1.60, *P* = 0.62). Finally, the occurrence of VAP correlated with less vasopressor-free days (− 5 [− 9; − 2] days) at day 28 and longer ICU (+ 13 [+ 9; + 15] days) and hospital (+ 11.5 [+ 7.5; + 17.5] days) LOS. These differences were observed in both subgroups (Table 3). Of note, when handling death as a competing event, the cumulative likelihood of ICU discharge over time was significantly lower in patients with VAP than in those without VAP (sub-distribution HR 0.57, 95% CI 0.44–0.74, *P* < 0.0001) (Additional file 1: Fig. S5).

## Discussion

The occurrence of a first episode of VAP was an independent predictor of death at day 90 in this cohort of 336 ARDS patients. This condition exerted a moderate impact on the PaO_2_/FiO_2_ ratio but correlated with a dramatic increase in MV duration, vasopressor use, and LOS.

The prognosis of VAP in ARDS patients managed with protective ventilatory settings has been the focus of merely two publications, both being ancillary analyses of randomized controlled studies conducted in the 2000’s [11, 12]. In the ACURASYS trial, VAP was linked with a substantial reduction in the number of ventilator-free days and ICU-free days at day 28 but not with a higher hazard of in-ICU death (adjusted odds ratio 1.41, 95% CI 0.83–2.39) [11]. Conversely, in the PROSEVA trial, VAP had a less pronounced effect on MV duration and LOS but was a strong risk factor for in-ICU death (aHR 2.21, 95% CI 1.39–3.52) [12]. In our population of unselected ARDS patients, VAP was associated with a more than twofold rise in MV duration and ICU LOS and a significant increase in the likelihood of death at day 90 (aHR 3.16, 95% CI 2.04–4.89). These discrepancies may result from case-mix variations. Notwithstanding its limited impact on oxygenation, VAP likely extends lung inflammation and alveolar damage as well as extra-respiratory organ dysfunctions. Indeed, in the present cohort, patients with VAP had less vasopressor-free days, VAP-related circulatory failure being associated with short-term mortality [30]. Hence, the higher mortality associated with VAP during ARDS could be primarily explained by prolonged exposure to the risk of dying due to delayed weaning from organ supports and increased ICU LOS, as proposed for the global population of critically ill patients receiving MV [6]. Interestingly, the cumulative likelihood of MV weaning did not differ between patients with and without VAP after adjustment on the competing risk of death, suggesting that VAP was rather a consequence than the cause of protracted MV duration.

Trends in PaO_2_/FiO_2_ and SOFA values following the diagnosis of VAP correlate with the hazard of clinical failure, pneumonia recurrence and death in the general population of intubated patients [31–33]. In ARDS patients, hypoxemia has been shown to resolve partly over the first days of adequate antimicrobial therapy [34, 35]; however, the consequences of VAP on oxygenation remain under-investigated in this population. In our cohort of patients with baseline criteria for severe ARDS in half of cases, VAP induced an only slight and transient alteration of gas exchanges, suggesting that the infectious process mainly affects lung areas with pre-existing consolidation and ventilation/perfusion mismatches. Interestingly, in a recent study including 255 patients (ARDS, 12.9%) with suspected VAP, PaO_2_/FiO_2_ values were poorly predictive of microbiological confirmation (area under the receiver operating curve 0.64, 95% CI 0.57–0.72) [36]. In addition, the limited correlation between VAP and ventilator-associated complications (VAC) or infection-related VAC (iVAC) partly results from a lack of sensitivity of the respiratory criteria for VAC/iVAC (that is, an increase in the FiO_2_ and/or PEEP levels after ≥ 2 calendar days of stability or decrease) for the detection of VAP [37]. Along this line, our data indicate that a decline in PaO_2_/FiO_2_ should not be considered as a pivotal trigger for VAP suspicion in patients with ARDS. Extra-respiratory organ failures could predict this diagnosis more reliably; indeed, SOFA values remained stable over the 2 days preceding VAP then significantly decrease thereafter, which may be ascribed to sepsis control and resolution under antimicrobial therapy.

Patients with COVID-19-related ARDS and those with ARDS from other aetiologies shared noteworthy similarities regarding VAP including pathogen distribution, time-courses of PaO_2_/FiO_2_ and SOFA values, and the cumulative likelihood of post-VAP extubation. PaO_2_/FiO_2_ values around VAP were lower in COVID-19 patients, a finding that corroborates the results of a recent work demonstrating worse oxygenation in these subjects—regardless of the occurrence of VAP—than in those with other ARDS despite comparable initial severity and respiratory system compliance after the third day of MV [38]. Nevertheless, VAP did not predict day-90 mortality in COVID-19 patients, contrary to what was observed in those with other ARDS, possibly due to a lesser extent of extra-pulmonary organ failures as suggested by the lower SOFA score values around VAP. An independent relationship between VAP and day-28 mortality has been reported in a multicentre cohort of critically ill COVID-19 patients (aHR 1.70, 95% CI 1.16–2.47); yet, in this study, the day-28 fatality rate was lower in patients with VAP than in those without ventilator-associated respiratory tract infection (25.9% versus 34.2%, respectively) [39].

This work has certain limitations. First, that the study was conducted in two ICUs of a single hospital may restrain its external validity; however, patients were managed according to current standards of care [20] and the epidemiological features of ARDS and VAP were concordant with those reported elsewhere [2, 11, 12, 40–43]. Second, diagnosing VAP is challenging in ARDS patients, especially in those with COVID-19 [5]; therefore, it cannot be firmly excluded that some patients with VAT were misclassified as having VAP though the divergent outcomes that we observed in the VAP and no VAP subgroups do not support this assumption, VAT being not associated with mortality in dedicated studies [44]. In addition, the SOFA score values at VAP onset in our cohort were higher than those previously reported in patients with VAT [45]. Third, the management of patients with severe COVID-19 has evolved since recruitment closing; while the early use of dexamethasone does not appear to increase the risk of VAP [46], other specific therapies such as anti-IL6 drugs might have modified the epidemiology of ICU-acquired infections [47]. Fourth, the relatively low number of COVID-19 patients could have precluded the detection of a significant effect of VAP on mortality in this subgroup. In addition, the prognostic impact of VAP might have been different in cohorts or settings with higher overall mortality rates. Finally, that prone positioning was used in only 22% of patients with non-COVID-19-related ARDS may have impacted the measured outcomes in this subgroup.

In conclusion, VAP is an independent predictor of day-90 mortality in ARDS patients. This effect was not observed in the COVID-19 subgroup; however, these analyses may have been underpowered. In both COVID-19 and non-COVID-19 patients, VAP exerts a limited effect on oxygenation but correlates with extended MV duration, vasoactive support, and LOS.

## Supplementary Information

**Additional file 1.** Additional tables and figures.

## Data Availability

The datasets used and/or analysed during the current study are available from the corresponding author on reasonable request.

## Abbreviations

ARDS: Acute respiratory distress syndrome
BMI: Body mass index
CI: Confidence interval
COPD: Chronic obstructive pulmonary disease
COVID-19: Severe coronavirus disease 2019
HR: Hazards ratio
ICU: Intensive care unit
LOS: Length of stay
MV: Mechanical ventilation
NMBA: Neuromuscular blocking agents
PaO_2_/FiO_2_: Partial pressure of oxygen/fraction of inspired oxygen ratio
PBW: Predicted body weight
PEEP: Positive end-expiratory pressure
PP: Prone positioning
SAPS 2: Simplified acute physiology score 2
SARS-CoV-2: Severe acute respiratory syndrome coronavirus-2
SOFA: Sepsis-related organ failure assessment score
VAP: Ventilator-associated pneumonia
Vt: Tidal volume
VV-ECMO: Veno-venous extra-corporeal membrane oxygenation
