# Supplementary material for: Clinical impact of ventilator-associated pneumonia in patients with the acute respiratory distress syndrome: a retrospective cohort study
Source: Ann Intensive Care. 2022 Mar 15;12:24. doi: 10.1186/s13613-022-00998-7 (PMC8922395; doi:10.1186/s13613-022-00998-7)
Supplement: Supplementary file 1 — Additional file 1. Additional tables and figures. [file 13613_2022_998_MOESM1_ESM.docx]

**ADDITIONAL MATERIALS**

**Clinical impact of ventilator-associated pneumonia in patients with the acute respiratory distress syndrome: a retrospective cohort study**

Marc Le Pape,^1,2^ MD; Céline Besnard,^1^ MD; Camelia Acatrinei,^1^ MD; Jérôme Guinard,^3^ MD; Maxime Boutrot,^2^ MD; Claire Genève,^2^ MD; Thierry Boulain,^1^ MD; François Barbier,^1^ MD PhD

**Author affiliations**

^1^Médecine Intensive - Réanimation, Centre Hospitalier Régional d’Orléans, Orléans, France ; ^2^Réanimation Chirurgicale, Centre Hospitalier Régional d’Orléans, Orléans, France ; ^3^Laboratoire de Bactériologie, Pôle de Biopathologies, Centre Hospitalier Régional d’Orléans, Orléans, France ; ^4^Centre d’Étude des Pathologies Respiratoires (CEPR), INSERM U1100, Université de Tours, Tours, France

**ADDITIONAL METHODS**

**Characteristics of the participating ICUs**

- Usual ventilator-bed capacities are 20 and 15 for the medical and surgical intensive care units (MICU/SICU), respectively, with only single-bed rooms and a 1:2.5 nurse-to-patients ratio in both units. These capacities transiently increased to 38 and 30 ventilator-beds during the study period (2 months, April-May 2020) due to the first COVID-19 wave, with a 1:3 nurse-to-patients ratio.
- Contact precautions are routinely applied in patients colonized or infected with multidrug-resistant bacteria.
- Full-barrier precautions are applied for COVID-19 patients.

**Bundles for prevention of ventilator-associated pneumonia**

- - Orotracheal intubation (rather than nasotracheal intubation) whenever possible
  - Semi-recumbent position >30°
  - Control of endotracheal tube cuff pressure (continuous or every 4-6 hours, target 20-30 cmH_2_O)
  - Oral care every 4-6 hours with saline 0.9%
  - Strict application of standard precautions, including alcohol-based hand hygiene
  - Endotracheal tubes with subglottic secretion drainage system are not routinely used in the participating ICUs
- Limitation of mechanical ventilation duration through (i) protocolized sedation in both participating ICUs, with nurse-driven titration of sedative agents (fentanyl and midazolam as first-line drugs; propofol, ketamine and/or dexmedetomidine as second-line drugs) every 2 to 4 hours according to a prescribed target of the Richmond Agitation-Sedation Scale, daily interruption except in patients with specified contraindication (e.g., acute brain injury or severe hypoxemia), and the use of enterally-administered oxazepam and/or haloperidol as step-down drugs when required, and (ii) management of mechanical ventilation weaning by both intensivists and specialized physiotherapists, with daily spontaneous breathing trial for patients meeting usual eligibility criteria and the routine preventive use of non-invasive ventilation ± high-flow nasal oxygen following extubation in patients with risk factors for weaning failure

**Policies for diagnosis and treatment of ventilator-associated pneumonia**

- Lower tract respiratory samples (either endotracheal aspirate, bronchoalveolar lavage or plugged telescopic catheter) are collected in every patient with suspected VAP before the start of a new antimicrobial therapy.
- Empirical single-drug or combination regimen are prescribed according to the patient’s risk factors for multidrug-resistant bacteria and de-escalation whenever possible once culture and susceptibility test results become available, in keeping with current guidelines (ATS/IDSA 2016 and ESICM/ESCMID/ERS 2017, available at [www.thoracic.org](http://www.thoracic.org) and [www.esicm.org](http://www.esicm.org), respectively).
- Follow-up respiratory samples are collected under therapy when required (*e.g*., persistent or worsening clinical, biological and/or radiological signs of pulmonary infection). The usual treatment duration is 7 days – longer durations may be decided by the treating physicians in case of immune deficiency, abscessed pneumonia or empyema, and evidence for clinical and/or microbiological failure at day 7.

**Table S1.** Full characteristics of the study population

|  | *Missing values* | **All patients with ARDS**  **(n = 336)** | **Patients with COVID-19-related ARDS**  **(n = 101)** | **Patients with ARDS from other causes**  **(n = 235)** | ***P* value** |
| --- | --- | --- | --- | --- | --- |
| **Unit of admission**  MICU  SICU | *0*  *0* | 254 (75.6)  82 (24.4) | 84 (83.2)  17 (16.8) | 170 (72.3)  65 (27.7) | NA |
| **Male sex** | *0* | 247 (73.5) | 73 (72.3) | 174 (74.0) | 0.79 |
| **Age, years** | *0* | 67 (57-74) | 67 (58-72) | 66 (55-74) | 0.73 |
| **BMI, kg.m^-2^** | *7* | 28.5 (25.0-32.6) | 29.4 (26.1-32.1) | 27.8 (24.1-32.9) | 0.13 |
| **Past or current smoking** | *0* | 132 (39.3) | 30 (29.7) | 102 (43.4) | 0.02 |
| **Chronic diseases**  Hypertension  Diabetes mellitus  COPD  Respiratory, other than COPD  Cardiac  Hepatic  Renal  Receiving chronic haemodialysis  Neurological  Immune deficiency  Solid cancer  Haematological malignancy  Others | *0*  *0*  *0*  *0*  *0*  *0*  *0*  *0*  *0*  *0*  *0*  *0*  *0* | 176 (52.4)  95 (28.3)  40 (11.9)  49 (14.6)  99 (29.5)  29 (8.6)  28 (8.3)  4 (1.2)  35 (10.4)  53 (15.8)  24 (7.1)  10 (3.0)  21 (6.2) | 55 (54.4)  40 (39.6)  6 (5.9)  17 (16.8)  25 (24.8)  2 (2.0)  5 (4.9)  1 (1.0)  6 (5.9)  11 (10.9)  3 (2.9)  3 (2.9)  5 (4.9) | 121 (51.5)  55 (23.4)  34 (14.5)  32 (13.6)  74 (31.5)  27 (11.5)  23 (9.8)  3 (1.3)  29 (12.3)  42 (17.9)  21 (8.9)  7 (3.0)  16 (6.8) | 0.63  0.003  0.03  0.50  0.24  0.003  0.19  1  0.08  0.14  0.06  1  0.63 |
| **Knaus score**  A-B  C-D | *0*  *0* | 308 (91.7)  28 (8.3) | 100 (99.0)  1 (1.0) | 208 (88.5)  27 (11.5) | 0.0008 |
| **Type of ICU admission**  Direct (Emergency department)  Transfer  Prior hospital LOS, days | *0*  *0*  *0* | 200 (59.5)  136 (40.5)  2 (1-5) | 52 (51.5)  49 (48.5)  3 (1-5) | 148 (63.0)  87 (37.0)  2 (1-5) | 0.05  0.82 |
| **Reason for ICU admission**  Acute respiratory failure  Impaired consciousness  Sepsis/septic shock  Cardiac arrest  Trauma  Scheduled surgery  Miscellaneous | *0*  *0*  *0*  *0*  *0*  *0*  *0* | 181 (53.8)  45 (13.4)  37 (11.0)  35 (10.4)  12 (3.6)  6 (1.8)  20 (6.0) | 101 (100)  0  0  0  0  0  0 | 80 (34.0)  45 (19.1)  37 (15.7)  35 (14.9)  12 (5.2)  6 (2.6)  20 (8.5) | NA |

**Table S1 (continued).**

|  | *Missing values* | **All patients with ARDS**  **(n = 336)** | **Patients with COVID-19-related ARDS**  **(n = 101)** | **Patients with ARDS from other causes**  **(n = 235)** | ***P* value** |
| --- | --- | --- | --- | --- | --- |
| **SAPS 2 at ICU admission** | *0* | 50 (38-67) | 40 (33-49) | 56 (43-71) | <0.0001 |
| **SOFA score at ICU admission** | *0* | 8 (5-11) | 5 (3-8) | 9 (7-12) | <0.0001 |
| **Lymphocyte count at ICU admission, mm^-3^** | *0* | 730 (447-1185) | 660 (470-870) | 780 (420-1310) | 0.03 |
| **Colonization with MDR-GNB**  All (pooled)  ESBLE | *0*  *0* | 79 (23.5)  64 (19.0) | 49 (48.5)  40 (39.6) | 30 (12.8)  24 (10.2) | <0.0001  <0.0001 |
| **ARDS aetiology**  COVID-19, no co-infection  COVID-19, bacterial or viral co-infection  Bacterial or non-SARS-CoV-2 viral pneumonia  Aspiration  Extra-pulmonary sepsis  Trauma  Haemorrhagic shock  Miscellaneous | *0*  *0*  *0*  *0*  *0*  *0*  *0*  *0* | 93 (27.7)  8 (2.4)  106 (31.4)  60 (17.9)  45 (13.4)  6 (1.8)  6 (1.8)  12 (3.6) | 93 (92.1)  8 (7.9)  -  -  -  -  -  - | -  -  106 (45.1)  60 (25.5)  45 (19.1)  6 (2.6)  6 (2.6)  12 (5.2) | NA |
| **ARDS and MV characteristics** ^1^  Lowest Vt, mL.kg^-1^ (PBW)  Highest PEEP, cmH_2_O  Highest plateau pressure, cmH_2_O  Highest driving pressure, cmH_2_O  Lowest PaO_2_/FiO_2_ ratio, mmHg  Highest PaCO_2_, mmHg  Lowest pH | *0*  *0*  *0*  *0*  *0*  *0*  *0* | 6.1 (5.8-6.6)  10 (7-13)  24 (20-27)  13 (10-16)  100 (74-163)  44 (40-52)  7.36 (7.25-7.4) | 6.0 (5.8-6.3)  12 (11-14)  26 (24-28)  13 (11-15)  91 (76-138)  43 (38-49)  7.36 (7.30-7.42) | 6.1 (5.7-6.8)  8 (6-12)  23 (18-26)  13 (10-16)  105 (74-172)  46 (40-55)  7.35 (7.22-7.39) | 0.02  <0.0001  <0.0001  0.91  0.17  0.0005  <0.0001 |
| **ARDS classification (Berlin definition)** ^1^  Mild  Moderate  Severe | *0*  *0*  *0* | 50 (14.9)  116 (34.5)  170 (50.6) | 11 (10.9)  30 (29.7)  60 (59.4) | 39 (16.6)  86 (36.6)  110 (46.8) | 0.09 |
| **ARDS-targeted therapies**  Prone positioning  Number of days  Nitric oxide inhalation  Neuromuscular blocking agents | *0*  *0*  *0*  *0* | 127 (37.8)  5 (2-11)  90 (26.8)  209 (62.2) | 75 (74.3)  8 (3-16)  46 (45.5)  86 (85.1) | 52 (22.1)  2 (1-5)  44 (19.1)  123 (52.3) | <0.0001  <0.0001  <0.0001  <0.0001 |

**Table S1 (continued).**

|  | *Missing values* | **All patients with ARDS**  **(n = 336)** | **Patients with COVID-19-related ARDS**  **(n = 101)** | **Patients with ARDS from other causes**  **(n = 235)** | ***P* value** |
| --- | --- | --- | --- | --- | --- |
| **Corticosteroids** ^1^  All (pooled)  Dexamethasone  Hydrocortisone  Methylprednisolone/prednisolone | *0*  *0*  *0*  *0* | 169 (50.3)  39 (11.6)  61 (18.2)  75 (22.3) | 53 (52.5)  34 (33.7)  6 (5.9)  13 (12.9) | 116 (49.4)  5 (2.1)  55 (23.4)  62 (26.4) | 0.64  <0.0001  <0.0001  0.006 |
| **Surgery during the ICU stay** | *0* | 48 (14.3) | 5 (4.9) | 43 (18.3) | 0.001 |
| **Decision to withhold or withdraw life-sustaining therapies** | *0* | 67 (19.9) | 13 (12.9) | 54 (23.0) | 0.04 |
| **Life-sustaining therapies during the ICU stay**  Invasive MV duration, overall, days Vasopressors  Renal replacement therapy  VA-ECMO  VV-ECMO | *0*  *0*  *0*  *0*  *0* | 11 (7-20)  280 (83.3)  85 (25.3)  7 (2.1)  15 (4.5) | 17 (10-26)  82 (81.2)  23 (22.8)  0  7 (6.9) | 9 (6-16)  198 (84.2)  62 (26.4)  7 (3.0)  8 (3.4) | <0.0001  0.52  0.58  0.11  0.16 |
| **Ventilator-associated pneumonia**  First episode  Prior MV duration, days  More than one episode | *0*  *0*  *0* | 176 (52.4)  7 (4-11)  59 (17.6) | 69 (68.3)  9 (8-13)  35 (34.6) | 107 (45.5)  6 (4-10)  24 (10.2) | 0.0001  0.01  <0.0001 |

*Table S1 footnote*

Data are expressed as number (%) or median (interquartile range).

MICU/SICU, medical/surgical intensive care unit; ARDS, acute respiratory distress syndrome; COVID-19, conoravirus disease 2019; BMI, body mass index; COPD, chronic obstructive pulmonary disease; MDRB, multidrug-resistant bacteria; LOS, length of stay; SAPS 2, simplified acute physiology score 2; SOFA, sepsis-related organ failure assessment; GNB, Gram-negative bacteria; ESBLE, extended-spectrum β-lactamase-producing Enterobacterales; MV, mechanical ventilation; Vt, tidal volume; PBW, predicted body weight; PEEP, positive end-expiratory pressure; VA/VV-ECMO, veno-arterial/veno-venous extracorporeal membrane oxygenation

^1^ First day with ARDS criteria

**Table S2.** Prior antimicrobial exposure, lower respiratory tract samples used for microbiological diagnosis, and pathogens responsible for VAP

| **Variables** ^1^ | **All patients with ARDS**  **(n = 176)** | **Patients with COVID-19-related ARDS**  **(n = 69)** | **Patients with ARDS from other causes**  **(n = 107)** |  |
| --- | --- | --- | --- | --- |
| **Antimicrobial exposure in the ICU before VAP**  Any antibiotic  BL/BLI  Duration, days  Non-antipseudomonal cephalosporins  Duration, days  Antipseudomonal cephalosporins  Duration, days  Carbapenems  Duration, days  Other β-lactams  Duration, days  Fluoroquinolones  Duration, days  Aminoglycosides  Duration, days  Anti-MRSA drugs  Duration, days  Metronidazole  Duration, days  Macrolides  Duration, days  Other antimicrobials  Duration, days | 167 (94.5)  101 (57.4)  5 (3-7)  92 (52.3)  4 (3-5)  15 (8.5)  4 (3-5)  22 (12.5)  5 (3-7)  24 (13.6)  5 (3-6)  7 (4.0)  5 (3-8)  19 (10.8)  1 (1-1)  39 (22.2)  4 (2-6)  23 (13.1)  4 (3-5)  72  3 (2-4)  5  8 (NA) | 67 (97.1)  20 (29.0)  4 (3-5)  55 (79.7)  4 (3-5)  8 (11.6)  4 (3-5)  9 (13.1)  5 (4-7)  9 (13.1)  5 (4-6)  1 (1.4)  6 (NA)  6 (8.7)  1 (1-2)  12 (17.4)  2 (2-3)  3 (4.3)  4 (NA)  48  2 (2-3)  2  4 (NA) | 100 (93.4)  81 (75.7)  5 (4-7)  37 (34.6)  4 (2-6)  7 (6.5)  5 (4-6)  13 (12.1)  4 (3-7)  15 (14.0)  5 (2-6)  6 (5.6)  5 (3-8)  13 (12.1)  1 (1-1)  27 (25.2)  4 (2-6)  20 (18.7)  4 (3-5)  24  3 (2-4)  3  8 (NA) |  |
| **Microbiological documentation**  Endotracheal aspirate  Bronchoalveolar lavage  Telescopic protected catheter | 135 (76.7)  27 (15.3)  14 (8.0) | 56 (81.2)  11 (15.9)  2 (2.9) | 79 (73.8)  16 (15.0)  12 (11.2) |  |
| **Pathogens responsible for VAP**  Enterobacterales  *Enterobacter cloacae*  *Escherichia coli*  *Klebsiella pneumoniae*  *Klebsiella aerogenes*  *Hafnia alvei*  *Serratia marcescens*  *Citrobacter koseri*  *Proteus spp*  Others  ESBL-producing isolates ^2^  *Pseudomonas aeruginosa*  *Stenotrophomonas maltophilia*  *Acinetobacter baumannii*  *Haemophilus* spp  *Staphylococcus aureus*  *Enterococcus* spp  *Streptococcus* spp  Others  Polymicrobial VAP ^3^ | 107 (60.8)  26 (14.7)  24 (13.6)  15 (8.5)  12 (6.8)  12 (6.8)  10 (5.7)  10 (5.7)  6 (3.4)  9 (5.1)  21 (11.9)  32 (18.2)  19 (10.8)  10 (5.7)  6 (3.4)  20 (11.4)  14 (7.9)  13 (7.4)  7 (4.0)  58 (32.9) | 41 (59.4)  11 (15.9)  7 (10.1)  7 (10.1)  6 (8.7)  6 (8.7)  7 (10.1)  3 (4.3)  2 (2.9)  0  12 (17.4)  12 (17.4)  6 (8.7)  6 (8.7)  2 (2.9)  8 (11.6)  4 (5.8)  7 (10.1)  1 (1.5)  19 (27.5) | 66 (61.7)  15 (14.0)  17 (15.9)  8 (7.5)  6 (5.6)  6 (5.6)  3 (2.8)  7 (6.5)  4 (3.7)  9 (8.4)  9 (8.4)  20 (18.7)  13 (12.1)  4 (3.7)  4 (3.7)  12 (11.2)  10 (9.3)  6 (5.6)  6 (5.6)  39 (36.4) |  |

*Table S2 footnote*

Data exposed as number (%) or median (interquartile range).

VAP, ventilator-associated pneumonia; COVID-19, coronavirus disease 2019; ARDS, acute respiratory distress syndrome; ICU, intensive care unit; ESBL, extended-spectrum β-lactamase; BL/BLI, β-lactam/β-lactamase inhibitor; MRSA, methicillin-resistant *Staphylococcus aureus*; NA, non appropriate

Anti-MRSA drugs indicate linezolide, daptomycin and glycopeptides.

Comparison between COVID-19-related ARDS and ARDS from other causes: ^1^ *P* = 0.64 (overall pathogen distribution); ^2^ *P* = 0.09; ^3^ *P* = 0.25

**Table S3.** Independent predictors of Day 90 mortality in patients with ARDS

|  | **Full Cox proportional hazards model** | |  | **Modified Cox proportional hazards model** ^1^ | |  |
| --- | --- | --- | --- | --- | --- | --- |
|  | **aHR (95% CI)** | ***P* value** |  | **aHR (95% CI)** | ***P* value** |  |
| Age, per 1-year increase | 1.02 (0.99-1.04) | 0.07 |  | 1.02 (0.99-1.04) | 0.09 |  |
| Weight | 0.98 (0.96-1.00) | 0.13 |  | 0.98 (0.95-1.00) | 0.13 |  |
| Chronic cardiac disease | 1.48 (0.96-2.29) | 0.07 |  | 1.61 (1.04-2.49) | 0.03 |  |
| Chronic renal disease | 2.11 (1.10-4.05) | 0.02 |  | 2.11 (1.09-4.09) | 0.03 |  |
| Chronic neurological disease | 1.32 (0.73-2.37) | 0.36 |  | 1.27 (0.72-2.27) | 0.41 |  |
| Cancer (solid or haematological) | 2.22 (0.71-6.95) | 0.17 |  | 2.5 (0.82-7.88) | 0.10 |  |
| Immune deficiency, any | 0.76 (0.30-1.87) | 0.54 |  | 0.71 (0.28-1.79) | 0.46 |  |
| SICU admission (versus MICU admission) | 0.71 (0.42-1.20) | 0.20 |  | 0.69 (0.40-1.7) | 0.17 |  |
| ICU admission for cardiac arrest | 2.00 (1.02-3.92) | 0.04 |  | 2.01 (1.01-4.01) | 0.04 |  |
| ICU admission for trauma or scheduled surgery | 0.39 (0.09-1.63) | 0.20 |  | 0.36 (0.08-1.50) | 0.16 |  |
| SAPS 2 at ICU admission, per 1-point increase | 1.02 (1.00-1.03) | 0.005 |  | 1.02 (1.00-1.03) | 0.02 |  |
| SOFA score at ICU admission, per 1-point increase | 1.04 (0.96-1.13) | 0.35 |  | 1.06 (0.98-1.15) | 0.16 |  |
| Lymphocyte count at ICU admission, per 1000.mm^-3^ | 1.01(0.83-1.23) | 0.91 |  | 1.05 (0.87-1.27) | 0.63 |  |
| pH value on the first day with ARDS criteria | 1.97 (0.34-11.36) | 0.45 |  | 2.50 (0.43-14.38) | 0.31 |  |
| COVID-19-related ARDS (versus ARDS from other causes) | 0.94 (0.54-1.66) | 0.84 |  | 0.71 (0.39-1.30) | 0.27 |  |
| Prone positioning (at least once during ICU stay) | - | - |  | 1.65 (1.01-2.70) | 0.04 |  |
| Steroid therapy | - | - |  | 1.12 (0.73-1.69) | 0.61 |  |
| **Occurrence of a first episode**  **of VAP** | **3.16 (2.04-4.89)** | **<0.0001** |  | **2.67 (1.72-4.14)** | **<0.0001** |  |

*Table S3 footnote*

ARDS, acute respiratory distress syndrome; BMI, body mass index; SICU/MICU, medical/surgical intensive care unit; SAPS 2, simplified acute physiology score 2; SOFA, sepsis-related organ failure assessment; COVID-19, coronavirus disease 19; VAP, ventilator-associated pneumonia

^1^ With prone positioning and steroids use during the ICU stay being forced into the model (*P* >0.20 in bivariate analysis)

**Figure S1.** Study flowchart

**653 patients with invasive MV >48h**

(1^st^ ICU stay during the inclusion period)

312 patients without ARDS

5 patients with ARDS excluded due to missing data

**336 patients (51.4%) with ARDS**

**included in the study**

**101 patients with**

**COVID-19-related ARDS**

**235 patients with ARDS**

**from other causes**

**69 patients (68.3%) with VAP**

**107 patients (45.5%) with VAP**

*Figure S1 footnote*

MV, mechanical ventilation; VAP, ventilator-associated pneumonia; ARDS, acute respiratory distress syndrome

**Figure S2.** Cumulative incidence of VAP

*Figure S2 footnote*

VAP, ventilator-associated pneumonia; ARDS, acute respiratory distress syndrome; COVID-19, conoravirus disease 2019; sHR, cause-specific hazard ratio (COVID-19-related ARDS versus ARDS from other causes, indicated with 95% incidence interval)

Day 0 indicates the date of intubation. Note that the curves are truncated at the time when every patients in each group had developed VAP, had died or had been weaned from mechanical ventilation.

**Figure S3.** Cumulative probability of survival after VAP onset in patients with COVID-19-related ARDS and ARDS from other causes

**
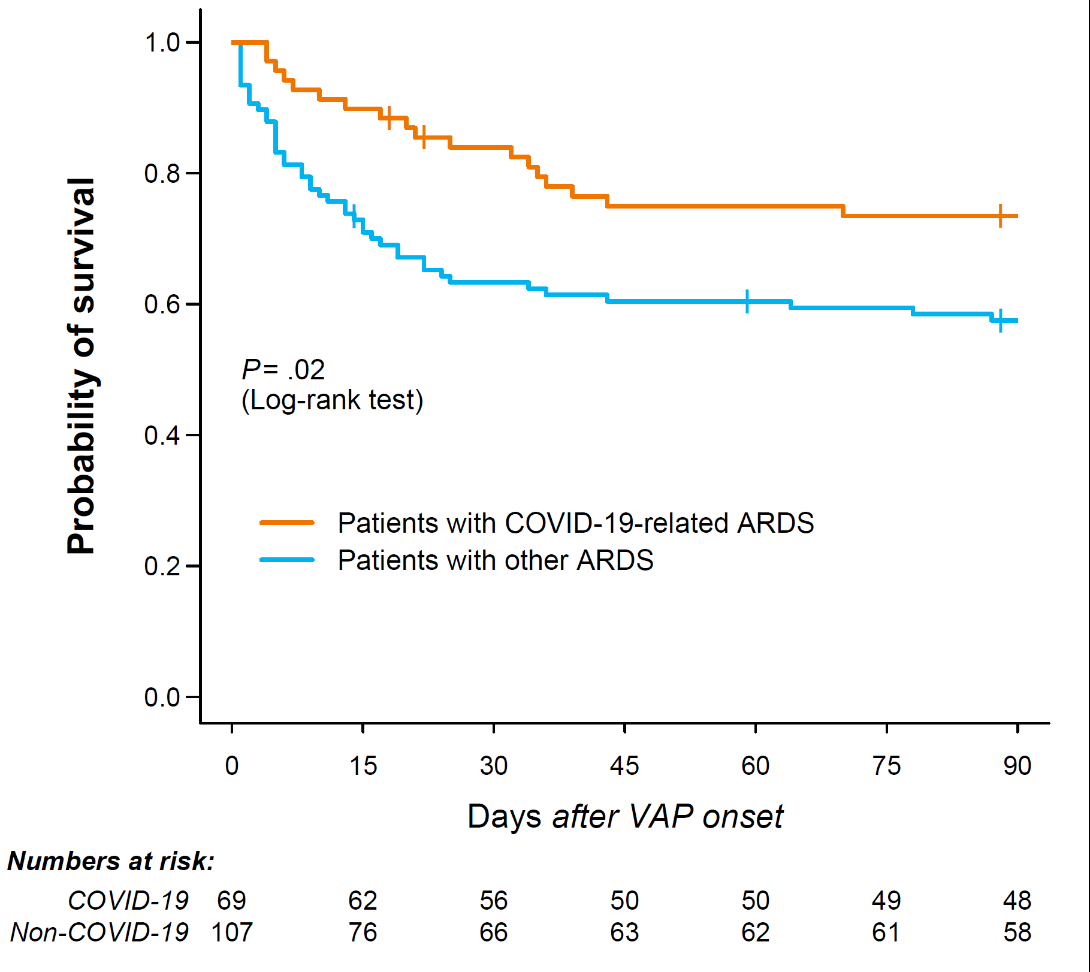
**

*Figure S3 footnote*

VAP, ventilator-associated pneumonia; COVID-19, coronavirus disease 2019; ARDS, acute respiratory distress syndrome

Day 0 indicates the date of VAP onset.

**Figure S4.** Trends in the level of PEEP in patients with VAP
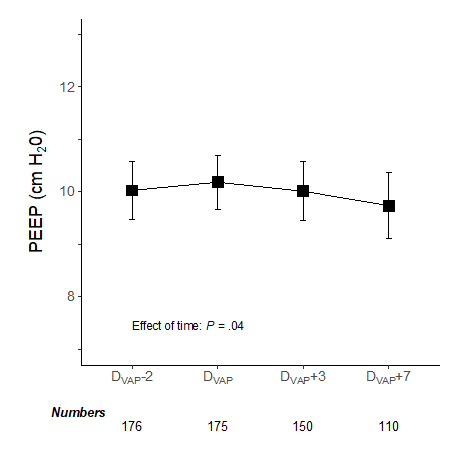

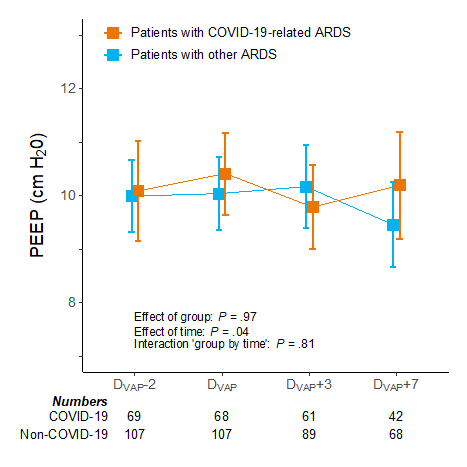


**B**

**A**

*Figure S4 footnote*

PEEP, positive end-expiratory pressure; VAP, ventilator-associated pneumonia; ARDS, acute respiratory distress syndrome; COVID-19, coronavirus disease 2019

Panels A, all patients with ARDS; panel B, patients with COVID-19-related ARDS versus patients with ARDS from other causes

**Figure S5.** Cumulative incidence of discharge alive from the ICU in patients with and without VAP

*Figure S5 footnote*

VAP, ventilator-associated pneumonia

Day 0 indicates the date of intubation. The correlation between the occurrence of VAP and the cumulative likelihood of discharge alive from the ICU was analysed handling VAP as a baseline characteristic (i.e., not a delay entry variable) and death as a competing event.
